# Towards translational research participation for nurses and midwives: a mixed method study

**DOI:** 10.1186/s12912-022-00818-0

**Published:** 2022-02-25

**Authors:** Gena Lieschke, Michelle Giles, Jean Ball, Se Ok Ohr, Vicki Parker

**Affiliations:** 1 Nursing and Midwifery Research Centre, Hunter New England Local Health District, Gate Cottage, James Fletcher Campus, 72 Watt Street, Newcastle, NSW 2300 Australia; 2 School of Nursing and Midwifery, The Faculty of Health and Medicine, University of Newcastle, Callaghan, NSW 2308 Australia; 3 School of Nursing and Midwifery, Faculty of Health, University of New England, Elm Avenue, Armidale, NSW 2350 Australia

**Keywords:** Translational research, Capacity building, Nursing, Midwifery, Embedding, Mixed-methods

## Abstract

**Background:**

Nurses’ and midwives’ participation in research has to date been highly variable and dependent on context and culture. A changing landscape that values and endorses research translation requires examination of who is participating in research and how, with an evaluation of current individual and organizational research capacity. The purpose of this study was to ascertain the existing research capacity amongst nurses and midwives in a large Local Health District in New South Wales, Australia to inform the development of a nuanced capacity building programme directed toward building a sustainable embedded research culture.

**Methods:**

A sequential mixed methods study design. Phase one, the exploratory phase, involved an online survey of all nurses and midwives (*n* = 8156) working in metropolitan, rural, and remote health services across the District. The survey measured research activity, skills, intention, value and relevance, organisational support, capability and culture, and research translation. Phase two, the explanatory phase, involved six focus groups with senior nursing and midwifery clinicians, educators, and unit managers, with discussion centred on the results of Phase one.

**Results:**

A total of 721 (88%) nurses and 95 (12%) midwives completed the online survey, 33 senior nurses and midwives attended focus groups. The nature and extent of research participation is variable across sites, individuals and clinical specialties. In many cases, interest and involvement in research is not sustained. Participants identified the need for greater incentives and structural support. Most important was the need for research to have tangible meaning for patients and clinical practice.

**Conclusion / implications for practice:**

Our findings suggest that translational research offers nurses and midwives the opportunity to engage in research in a way that is meaningful to their practice and their aspirations. Greater emphasis is needed on the development and enactment of context specific nursing and midwifery research agendas and implementation research skills.

## Background

Over the last decade, the push to ensure research is translated into tangible, timely outcomes for patients, health professionals, health care organizations, and policymakers has gained momentum. Translation of research requires that much of the doing of research takes place in the context of practice, where nurses and midwives need to be engaged with, and take a lead role in research that impacts on their work, and the outcomes of their care [1–4].

Numerous reviews of nurses’ and midwives’ participation in research have been conducted over the last two decades [5–7]. The shifting focus of these reviews chronicles a changing culture and growing activity, with a shift from research being done by nurse academics to integration into the role and expectations for clinical nurses’ and midwives’ [8, 9].

A systematic review of research activity and capacity building in the practice of clinical nurses conducted by Lode et al. [6] identified three critical features; failure to ensure research quality and standards, lack of knowledge about how to increase research capacity, culture and collaboration, and how to increase and organize research utilization. Research capacity building is a complex endeavor requiring sustained multi-level engagement. Research capacity building requires the development of knowledge and competence, together with policy, infrastructure and teams with the strong stewardship of committed leaders [6, 8].

Despite the growing understanding of the role of research in ensuring best practice outcomes, the engagement and activity of nurses and midwives in research appears to be limited. More specifically, the way in which growing emphasis on translational research has impacted the roles and activity of nurses’ and midwives’ more broadly but also in specific contexts has not been examined. Chen et al. [8] suggest that lack of clarity around key concepts together with lack of appropriate tools and the need for consideration of context have been deterrents to understanding nurses’ research participation.

In light of these concerns and changes, this study sought to examine clinical nurses’ and midwives’ views about research, the nature of their engagement in research, and the contextual factors that inhibit or promote participation in research within a large regional Local Health District (LHD) in New South Wales (NSW), Australia.

The material and organizational barriers to nurses and midwives undertaking research are well described [6–14]. The need for research capacity building is well recognized and attributes for successful models proposed [15, 16]. However, models need to be tailored to context based on comprehensive knowledge of the workforce, opportunities and limitations of the context in which the capacity building will take place. Further, ongoing assessment is necessary to ensure and assess the impact of research activity.

Understanding the current research activity and capacity of nurses and midwives, together with organizational supports and culture is essential to developing a productive and sustainable research environment [17]. Without comprehensive knowledge of the state of play in terms of who is doing research and where, where interests and opportunities lie, and what level of support is available, it will not be possible to build and enact a meaningful research framework and strategies that will optimize nursing and midwifery research input and outcomes into the future.

This paper reports the findings of phases one and two of a study that sought to examine nurses’ and midwives’ research capacity, activity, and interest across a large regional LHD in NSW, Australia. Changes in government policy arising in relation to health research have highlighted the imperative for research to have outcomes and impact within the LHD. This change has enabled expansion and resourcing of a District Translational Research Unit responsible for the oversight and direction of research conducted within the LHD, and the revision of the role of the LHD’s Nursing and Midwifery Research Centre (NMRC).

The NMRC’s primary purpose is to build research capacity amongst nurses and midwives across the LHD, together with leading research on priority clinical issues. The study results will inform the strategic work of the Centre and help determine ways in which nurses and midwives can contribute to LHD-wide multidisciplinary, translational research initiatives.

## Methods

### Aim

The aim of the study was to identify existing research capacity and capability, and requirements necessary to promote, support and enable an embedded sustainable nursing and midwifery research culture within the LHD.

### Research question

What are nurses’ and midwives’ views about research, the nature of their engagement, and the contextual factors that inhibit or promote their participation?

### Design

A sequential mixed methods study guided by Interpretive Description. Interpretive description is a qualitative approach that focusses on the achievement of an actual practice goal drawing on evidence from all available sources [18]. The purpose of this research is to gather information that will support the improved research participation of nurses and midwives, increase the amount and quality of research undertaken by them, and to ensure that research has meaning for clinicians and patients.

Phase one involved an online survey sent to all nurses and midwives working in clinical, education and management roles across the LDH. Phase two involved focus groups with nurses and midwives most likely to be actively engaged in research, including those with research required in their role descriptions, and those with accountability for research within their clinical unit or speciality.

### Data collection

#### Phase one - survey

Information about the study was presented to senior nursing and midwifery service managers at leadership forums. Service managers disseminated study flyers and information to all senior nurses and midwives within their respective jurisdictions across the LHD. Following initial advertisement, invitations to participate in the study, along with a link to the self-administered survey was sent to all nurses and midwives employed in the LHD (*n* = 8156) via their staff email. The survey was open for 6 weeks February/ March, 2017, with reminders sent weekly. Participation in the survey was voluntary, no identifiable information was collected, and participant anonymity was protected.

The survey included previously validated instruments, identified from the literature (Table 1), which measured individual research skills/aptitude (4 items), perceived individual research intentions (3 items), perceived individual research capacity (15 items), as well as factors related to perceived research value (7 items), perceived research relevance (9 items), perceived organizational culture and capability (19 items), perceived organizational support (3 items), and individual respondent characteristics (3 items) [19–22]. In addition, a seven-item translational research domain was developed after deliberation and critical appraisal by the research team [23]. Other questions measured research activity. The survey was piloted with a sample of 10 senior nurses prior to wider distribution. Permission was given by the original authors to use all or part of their measurement tools. Survey constructs and definitions are outlined in detail in Table 1.

**Table 1:** Survey constructs (Modified from Parker et al., 2017) [23]

	Definition	Measurement Tool
**Individual Domain**		
Perceived individual research intention	Individual’s intent to engage with research activities and opportunities in order to inform their practice.	Research and Development Culture Index (R & D Culture Index) (Watson et al., 2005) [19]
Perceived individual research capacity	Individual skill level across a variety of research related activities from finding the literature through to dissemination of findings	Research and Development Culture Index (R & D Culture Index) (Watson et al., 2005) [19] Research and Capacity Culture Tool (RCC Tool) (Holden et al., 2012) [20]
Perceived research relevance	Importance individual places on research for practice improvement and significance in daily work, relevance to profession and relevance to education	Nursing Research Questionnaire (NRQ) (Corchon et al., 2010) [21]
Perceived research value	Value and impact of research in practice and on their profession	Nursing Research Questionnaire (NRQ) (Corchon et al., 2010) [21]
Perceived translation of research into practice	Explores whether research is collaborative between clinicians and researchers, is directed by strategic priorities, improves patient and organizational outcomes through sustained practice change and used to evaluate interventions.	Developed and validated by the research team (Parker et al., 2017) [23]
**Organizational Domain**		
Perceived organizational support	Degree of organizational support and opportunity for, and application of research in your team or service	Research and Capacity Culture Tool (RCC Tool) (Holden et al., 2012) [20] Queensland Health Practitioner Research Capacity Survey (Queensland Health Practitioner Research Capacity Survey) [22]
Perceived organizational culture and capability	Degree of research related resources, planning, leadership, opportunities, consumer involvement, and quality monitoring and expert advice.	Queensland Health Practitioner Research Capacity Survey (Queensland Health Practitioner Research Capacity Survey) [22]

#### Phase two - focus groups

Findings from phase one informed the second explanatory phase of the study that included a series of focus group interviews held in rural and regional sites in the LHD. Focus groups were advertised at senior service manager and clinician meetings, and information and consent forms disseminated by service managers within their respective services. Upon receipt of signed consent forms respondents were contacted to schedule focus group participation. Recruitment to the focus groups was purposive. Inclusion criteria was based on participants experience in, or responsibility for conducting research within their clinical, education or managerial role. Care was taken to ensure equal representation across geographical location and professional roles. Six focus groups (approximately 60 min duration), three in rural, and three in metropolitan sites were facilitated by experienced qualitative researchers (GL, MG, VP). A total of 33 participants attended one of the six focus group interviews.

Focus group questions were asked about nurses and midwives’ involvement in research, perceptions related to the value and impact (potential or realized) of nursing and midwifery research within the LHD, the supports required to integrate research activity into clinical roles and aspirations to conduct research.

### Data analysis

Survey data were analyzed using a descriptive summary to produce a demographic profile of nurse and midwife respondents. Confirmatory factor analyses were conducted on constructs from the previously validated tools and the newly created tool. Cronbach alphas (to assess internal consistency) were calculated on the factors. Outcomes for analysis were selected based on factor analysis showing good internal consistency and validity for the factor. Two binary outcomes, based on single questions in the survey, were also selected as outcomes: ‘Desire to participate in research (Research Intention)’, ‘Participation in research in last 5 years’.

Covariates of interest were: ‘age’, ‘current position’, ‘current employment status’, ‘highest educational qualification’, ‘specialist area of work’ and ‘location of workplace’.

Univariate regressions were conducted for each covariate with each outcome. Covariates with a *p*-value < 0.20 and a relevant effect size in the univariate analyses were included in the multivariable model for each outcome. Two logistic and four linear multivariable regressions were conducted on the selected response outcomes to identify associations.

Results of regressions are presented and interpreted as follows:

- *Categorical outcome with categorical covariate:*

Odds ratios represent the change in the odds that the outcome will occur for a level of a particular covariate, compared to the odds of the outcome occurring for the reference value of that covariate.

- *Categorical outcome with continuous covariate:*

Odds ratios represent the change in the odds that the outcome will occur, for each one unit increase in the score of that covariate.

- *Continuous score outcome with categorical covariate:*

Least square (LS) mean scores of the outcome are presented for each level of the categorical covariate. The probability indicates whether the mean is significantly different to the mean of the reference level of the covariate.

- *Continuous score outcome with continuous covariate:*

Regression coefficients are interpreted as the amount of increase in the outcome score for each one unit increase in the covariate, when all other covariates are held constant.

Once transcribed, each focus group transcript was coded into themes and worked into a descriptive account. Common themes were identified across transcripts to provide an overall account. This was done independently by three researchers (GL,VP, MG), compared and final themes confirmed [24]. Particular attention was focussed on convergent and divergent responses within and across rural and metropolitan groups and between participants with more or less flexible roles eg, clinical nurse consultants (CNC) and clinical midwifery consultants (CMC) versus registered nurses (RN) and clinical nurse specialists (CNS) and clinical midwife specialists (CMS). Themed data were then compared with survey findings and integrated into the final report.

Rigor of the study was ensured through consistency of questions and theoretical concepts across data collection methods. Qualitative data analysis rigor was demonstrated through keeping an audit trail of coding and theme development by individuals which was then compared and verified across researchers.

### Ethical considerations

Ethics approval to conduct the study was granted by LHD Human Research Ethics Committee (HREC) (Reference number 15/12/16/5.09).

## Results

The results section firstly presents the survey respondents demographics and the factor analysis of the seven factors explored in the survey (Table 1). Secondly, survey and focus group data are combined to present the findings based on three overarching themes; Research activity-outlining current and previous research involvement and factors affecting level of involvement, Views and attitudes – perceptions of research relevance, value and translation into practice and Preparedness for research – at an individual and organizational level, related to capacity, supports, opportunities, culture and capability.

### Demographics

A total of 721 (88%) nurses and 95 (12%) midwives completed the online survey in Phase One, representing 10% of the total LHD Nursing and Midwifery workforce (See Table 2). Survey respondents were representative for gender and slightly older than the total workforce. There was equal representation across rural and metropolitan respondents (51% v 49%). The proportion of respondents employed within the LHD for more than 10 years was higher than in the total workforce (60% vs 39%). A higher proportion of more senior nurses and midwives (CNC/CMC and CNS/CMS) participated in the survey. The primary practice environments of survey respondents were; acute (46%), primary (27%), emergency (8%), rehabilitation care (6%), and preventative health (5%). The clinical specialities represented included; medical and surgical nursing, critical care, perioperative, paediatrics, mental health, family & child health, midwifery, rehabilitation, community and aged care.

**Table 2:** Survey respondent demographics

		Survey respondents (*N* = 816)	Total Workforce (*N* = 8156)	
Age	Mean (95% CI)	47 (46, 48)	45 (47, 48)	
	Median	49	46	
	Range	22–69	18–80	
		N (%)	N (%)	
Gender	Female	725 (90%)	7504 (92%)	
	Male	77 (10%)	652 (8%)	
Employment status	Permanent	720 (90%)	6933 (85%)	
	FTE 1.0	432 (54%)	3344 (41%)	
Length of time in LHD > =10 yrs		475 (60%)	(39%)	
Location	Rural/remote	403 (51%)	3099 (38%)	
	Metropolitan	387 (49%)	5057 (62%)	
Employment classification	RN/RM	357 (44%)	5397 (66%)	
	EN	68 (8%)	1186 (15%)	
	CNS/CMS	118 (15%)	767 (9%)	
	CNC/CMC	130 (16%)	226 (3%)	
	NP	19 (2%)		
	Clinical Educators	46 (6%)	23 (< 1%)	
	Managers	73 (9%)	366 (5%)	
	Other	17 (2%)	191 (2%)	
		Already attained	Currently undertaking	Planning to undertake
Highest Qualifications	PhD Prof Doc	3 (< 1%)		3 (< 1%)
	Masters (Research)	18 (2%)	2 (< 1%)	12 (1%)
	Masters (Course work)	137 (17%)	43 (5%)	61 (7%)

*RN/RM* Registered nurse/registered midwife, *EN* Enrolled Nurse, *NP* Nurse Practitioner; Clinical Educators includes Clinical nurse educators (CNE), clinical midwife educators (CMS), nurse educators (NE) and Midwife educators; and Managers include nursing unit managers, midwifery unit managers, nurse managers and midwifery managers

In Phase Two, a total of 33 participants attended six focus groups conducted across the LHD. Three groups were conducted in a metropolitan setting (*n* = 13) and three in a rural setting (*n* = 20). Focus group participants at the metropolitan centre were represented by; CNC’s from clinical specialities including mental health, rehabilitation, cancer, aged care, pain management and population health (*n* = 8), clinical nurse educators (CNEs) from paediatrics and general surgery (*n* = 3), and CNS’s from Anaesthesia, Midwifery and Paediatrics (n = 3). In contrast, rural focus group participants were primarily CNE (*n* = 7), along with nurse unit and quality managers (*n* = 5), CNS (n = 7) and one CNC, with representation across a broad range of speciality areas.

### Factor analysis

After assessing the internal validity of the scores, four scores were selected as outcomes for analysis: ‘Perceived Individual Research Capacity’, ‘Perceived Translation of Research into Practice’, ‘Perceived Organizational Support’, and ‘Perceived Organizational Culture and Capability’ as well as the two previously defined binary outcomes: ‘Perceived Research Intention’, ‘Participation in research in last 5 years’ (see Table 3).

**Table 3:** Factor analysis results

FACTORS	Cronbach alpha	Mean	N	Items/ Scale
Perceived Organizational Culture and Capability	0.976	4.30	534	19 / 1–10
Perceived Organizational Support	0.862	2.88	574	3 / 1–5
Perceived Individual Research Capacity	0.959	4.13	700	15/ 1–10
Perceived Translation of Research into Practice	0.860	3.30	619	7 / 1–5
Perceived Research Intention	0.832	3.12	701	4 / 1–4
Perceived Research Value	0.762	3.09	624	7 / 1–4
Perceived Research Relevance	0.672	2.93	625	9 / 1–4

### Research activity

The amount and nature of research activity were examined in both the survey and focus groups, along with who was leading the research. Approximately one-third of survey respondents (29%, *n* = 230) indicated they were currently involved or had previously been involved in research. Of these, one fifth (*n* = 45) reported participating in five or more projects in the previous 5 years. Over half of respondents (52%, *n* = 424) indicated they had a desire to become involved in the research. However, 48 (21%) of those who had been involved in research reported they no longer wanted to be involved.

Survey respondents reported involvement across all study types with clinical trials and qualitative studies being the most common. In total, 126 (15.5%) respondents reported being involved in research led by nurses or midwives. Although some respondents were involved in nurse-led research, more than half of the research in which they participated was led by others (doctors, academics, scientists, drug companies). Nurses were most likely to lead qualitative or mixed methods studies.

Several covariates were significantly associated with respondents who had been involved in research in the past 5 years (see Table 4). CNC/CMCs were more likely than RNs/RMs to have been involved in research (OR = 7.6 [CI 3.0, 19.1], *p* <  0.001). Those working in mental health were 6.7 times more likely than those working in midwifery to have been involved in research (OR = 6.7 [CI 1.9, 23.6], *p* <  0.01), whilst respondents employed in rural and remote services were less likely than their metropolitan counterparts to have participated in research (OR = 0.4 [CI:0.2, 0.8], *p* < 0.01). Respondents reported being involved in a range of project types. The roles most often assumed by nurses and midwives participating in research ranged from lead investigators (28%), to clinical trial coordinators (10%), and research assistants (24%). There was no difference in research intention between employment classifications, qualifications, locations of employment.

**Table 4:** Associations between research factors and covariates of interest

**Survey item** **(Binary outcome)**	**Covariate**		**Odds ratio (95% CI)**	***p*-value**
**Previous Involvement in Research**				
	Employment Classification (RN/RM compared to CNC/ CMC)		7.6 (3.3, 19.1)	< 0.001
	Speciality (Midwifery compared to Mental Health)		6.7 (1.9, 23.6)	< 0.01
	Position location (Metropolitan compared to Rural)		0.4 (0.2, 0.8)	< 0.01
**Factor** **(Continuous outcome)**	**Covariate**		**Mean score (95% CI)**	***p*-value**
**Perceived Translation of Research into Practice**				
	Medical specialty compared to Midwifery		24.3 (23.3, 25.2)	0.02
	Critical care compared to Midwifery		24.6 (23.4, 25.9)	0.02
	Palliative care compared to Midwifery		24.6 (22.5, 26.7)	> 0.05
	Perceived Value of Research		0.18 (0.03, 0.33)	< 0.02
	Perceived Organizational Support		0.47 (0.31, 0.62)	< 0.01
	Perceived Organizational Culture & Capability		0.01 (0.00, 0.02)	< 0.01
**Perceived Individual Research Capacity**				
	Highest qualification	PhD, Prof Doc, Research Masters	85.8 (74.9, 96.7)	< 0.01
		Masters Course Work	72.7 (67.4, 78.0)	< 0.01
		Certificate	56.3 (49.4, 63.2)	0.03
		No research ≤5 yrs	2.53 (1.11, 3.94)	< 0.01
**Perceived Organisational Culture and Capability**				
	Metropolitan vs Rural/remote		0.55 vs 0.45	0.02
	Perceived Translation of Research		1.35 (0.3, 2.40)	0.01
	Perceived Organisational Support		7.95 (6.06, 9.84)	< 0.01
**Perceived Organizational Support**				
			**Least square Mean score (95% CI)**	***p*-value**
	Employment classification	CNC/CMC	9.5 (8.8, 10.2)	< 0.01
		Nurse/Midwife Unit Manager	9.3 (8.3, 10.3)	0.05
			**Regression coefficient (95% CI)**	***p*-value**
	Perceived Research Relevance		0.16 (0.09, 0.23)	< 0.01
	Perceived Translation of Research		0.11 (0.07, 0.16)	< 0.01
	Perceived Organizational. Culture & Capability		0.01 (0.01, 0.02)	< 0.01

The diversity of research involvement across specialty, professional classification and geographical location reported in survey results, was echoed in focus group discussion. Metropolitan focus group participants reported higher levels of research involvement than rural participants and were more likely to recount being involved in nurse led, or multidisciplinary team research projects. CNCs recounted greater research involvement than any other group. Research activity was most frequently reported by respondents from mental health, population health, cancer and aged care, and reflect a higher proportion of respondents from these specialties having received or currently undertaking research higher degrees.

Further, focus group participants recounted being exposed to a range of research projects, including qualitative and quantitative studies, small scale service evaluation and clinical trials conducted largely by medical colleagues, commissioned researchers and academics, as well as large multi-centre clinical drug and device trials.

A lot of research in our service is medically focused and medically led. (FG3_Metropolitan CNE)

If you go to the ward, there seems to be a million other people doing research. (FG1_Metropolitan CNC)

The nature and extent of research participation is variable across sites and individuals, according to opportunity, and in many cases interest and involvement in research is not sustained.

*I've struggled to bring nurses along with me. They express interest and then it doesn't go beyond that. The active involvement and initiative to do the work is not sustained and you can't keep going to people and say, "Are you going to do this", so you just end up doing it yourself*. *(FG3_ Metropolitan CNC)*

Only a small number of metropolitan CNCs described conducting their own research. These clinicians recounted undertaking projects that were patient -oriented and solution focused and that involved multidisciplinary team participation. Additionally, a small number reported conducting independent research, most often to fulfill formal research training requirements, and for two senior clinicians, leading clinician team-based research. One CNC described embedding research into everyday work practice.

*We have been able to build research into everybody's work as a normal part of business. Everybody is on a research project of some sort and it’s stuff that we are doing, it's not separate stuff.* (*F*G2_*Metropolitan CNC*)

Focus groups participants identified three key forms of participation; their own need and motivation to improve health outcomes, State or District wide initiatives rolled out at a local level, and joining research groups usually led by doctors or career researchers. A significant amount of research was conducted as research higher degree studies, with varying degrees of connection to practice environments.

### Views and attitudes toward research

Views and attitudes toward research were canvassed in the online survey, and further explored during focus group discussions. Survey respondents reported moderate levels of perceived research relevance (item mean = 2.93 out of 5) and perceived research value (item mean = 3.09 out of 5) (Table 3). Perceived research value scores were significantly higher for those in senior clinical roles when compared to manager scores (mean scores 22.4 and 21.5 out of 36 respectively, *p* = 0.02). Metropolitan respondents scored significantly higher than their rural counterparts in both perceived research relevance (*p* = 0.03) and perceived research value (*p* < 0.01) (Table 4). There were no differences identified across the different specialty areas for both factors.

It is not surprising that as senior clinicians, most focus group participants expressed the view that research is important and were keen to include research into their role, however they did not feel that their positive view was shared more generally by colleagues.

I think it's really important that nurses are involved in research. It is no good just doctors doing research, we need nursing research to keep us moving into the future and it's really important that we grab hold of that…//.. There is definitely the opportunity to do things, it’s just the commitment. (FG5_ Rural CNS)

We get involved in a lot of area wide research projects. You have to sell the projects and a lot of the time it is not well received by staff..//.. Staff see it as extra work and confusing work. (FG5_ Rural Midwifery Unit Manager)

Further, focus groups participants reported a persistent view amongst many clinicians that research was a secondary non-essential activity.

Research is one of those things that is nice to do, we don't have to do it. And that research participation is daunting and difficult. Research really freaked me out for a long time. It was something that was too hard. (FG1_ Metropolitan CNC)

They also described their experience of numerous projects that failed to bring about change and as a consequence they struggle to get support from managers for their research;

I think we tend to do a lot of research and quality improvement that run their course and don't actually change anything. I'm also starting to find a lot of managers are change wary and if we go to them wanting to do a project, it's a flat out "no". (FG3_Metropolitan CNC)

Those in Metropolitan areas showed no difference in their perception that research was being translated into practice than those in rural/remote areas. Those working in the specialties of Medical, Critical care and Palliative care had the highest perceptions that research was being translated into practice than other specialty areas (LS mean scores: 24.3, 24.6 and 24.6 out of 35, respectively), with medical and critical care specialties both being significantly higher than Midwifery (*p* = 0.02 for both).

Across all focus groups, participants highlighted two nurse-led (LHD-wide) implementation projects that had been impactful. These projects have been instrumental in helping make visible the link between research and practice, for example by reducing indwelling urinary catheter use and improving outcomes for stroke patients.

Our primary purpose is to provide healthcare services, so with translational research it needs to be about something that is important and also something that is going to stay in care, some of the example like the urinary tract infection ones, that's practice that has stayed with us. Whereas other research … // … can become a casualty regardless of how successful the project is. (FG5_ Rural CNS)

However, negative views of research were expressed by the participants with associated perceptions that research created extra and often unnecessary work, and that it is not relevant or nuanced enough to respond to the contextual demands particularly in rural services.

You can sell it till the cows come home, but if it's not relevant to this (rural) site, you won't get collaboration from staff because they can't see the value in it, it won't get off the ground. (FG4_ Rural CNS)

Without relevance to and derivation from practice, research was seen as pointless, without direct impact on nursing it was seen as someone else’s research.

Nurses and midwives are interested in doing research that comes from clinical care..//..things that matter at ward level..//..we collect so much data and report all sorts of things, but most of it is unrelated to what we are doing. If we are asked to collect data, we should be doing something with it. Ward nurses get really disheartened because they think what's the point. (FG3_ Metropolitan CNC)

One participant described how doing small scale incremental research often works best for nurses and midwives.

Sometimes the most effective nursing research is just small pieces done well that can get completed. Then you do another small piece and build like a jigsaw puzzle. Just small pieces and then over time build a picture. (FG2_Metropolitan CNC)

With success and tangible results clinicians begin to see the value and are more willing to become engaged.

### Preparedness for research

Preparedness was examined via the survey at individual and organizational levels. From an individual perspective, perceived individual research capacity was measured using 15 items outlined in Table 1. Respondents rated their perceived research capacity highest for being able to find and critically review the literature, and lowest in submitting ethics and preparing grant applications for funding.

Focus group participants identified similar research activities for which they needed support;

Nurses need practical support in areas where they are not expert, and that’s writing grant applications, ethics submissions..//.. Because there is a huge gap. (FG2_ Metropolitan CNC)

In our area we struggle a little bit with research and there are quite a few of us wanting to get involved in research, but don't know where to start..// … It is very difficult to find opportunities and we are sort of struggling to find help. (FG1_ Metropolitan CNE)

From an organizational perspective, respondents reported a low to moderate perception of available organizational research supports and opportunities across the LHD (item mean score of 2.88 out of 5) (Table 3), with rural respondents indicating slightly less perceived support than Metropolitan respondents. CNC/CMCs perceived organizational supports as higher than RN/RMs (mean score = 9.5 [95% CI: 8.8, 10.2], *p* < 0.01). Regression analysis demonstrated a significant positive association between the perceived organizational support and perceived research relevance (p < 0.01), perceived Research Translation (p < 0.01), and perceived organizational culture and capability (p < 0.01) (Table 4).

CNCs and educators in the focus group were frustrated by the lack of importance and resources given to research by the organization;

There is a lot of good will, a lot of willing participants, but there are a lot of obstacles and barriers. (FG1_ Metropolitan CNC)

I think managers would be supportive, but it's finding the time to do it. Managers are caught between wanting people to be able to do it, but then being able to back fill them to allow them to go. (FG2_ Metropolitan CMS)

A second organizational perspective was perceived organizational research culture and capability. Scores were generally low with a median score below 4 (out of 10) for all items and inconsistent across all positions. The CNC/CMC group perceived organizational culture and capability to be higher than other groups, but moderately so, highest in palliative care and lowest in aged care specialty areas. Regression analysis demonstrated a significant positive association with perceived Research Translation of Research into Practice (*p* = 0.01) and perceived organizational support (*p* < 0.01).

In a complex and busy work environment, without the resources and structural levers that generate and support research activity there is little incentive for research initiation and participation, and hence the opportunity to achieve sustainable change.

There is no time for research and there is no budget for it either. Everyone is just trying to meet their KPIs [key performance indicators] and research isn't in their KPIs. (FG3_ Metropolitan CNS)

What we need is a structured set up that is integrated into the Executive Leadership Team. We need leaders that support the activity at this level, leaders who have a real voice at the executive level, who recognizes research is part of nursing…// … someone who has the legitimacy and power to ask staff to be accountable for research. (FG2_ Metropolitan CNC)

## Discussion

Our findings indicate that research expertise and participation is variable across sites and roles, and dependent on recognition of research as integral to practice, and support in the form of time and access to resources. There is a strong view held by some clinicians that research is important, and also some tension associated with lack of support given to nurses and midwives to undertake research as part of their role. Previous studies about nurses’ perceptions of research have indicated that research is not perceived as nursing work, is of secondary importance behind patient care, and that being involved in research is seen as a pathway to move away from bedside nursing [7, 9]. Barriers to nurses undertaking research are well documented, with lack of knowledge, time and support reported as consistent impediments [9–14].

In line with this previous research, there is also a persistent view amongst our respondents that there is no time to conduct research and there is a need to focus on practice rather than research [10–15]. Our findings are similar to those of other studies conducted with nurses and midwives and health care professionals generally [10, 11, 15].

Nurses and midwives in our study indicated they are more likely to engage with research if they consider that it will improve patient outcomes, and when they are engaged as key contributors, supported and recognized for their efforts [10, 14]. Without these key elements research is viewed as an imposition, as too hard and too time-consuming. Participant’s accounts portray a research context characterized by marked differences across sites in terms of the amount of research being conducted and the degree of integration of research in everyday work practice. Consistent with the findings of Schmidt et al. [15], rural clinicians in this study are less likely to be involved in research and have less organizational support for research. Similar to the findings of the study by Scala et al. [13] and Caldwell et al. [10], there is strong indication that there is valuing of research, a shift toward translational research and growing involvement of nurses and midwives. This shift is accompanied by an increase in the numbers of nurses and midwives undertaking research higher degree studies. Of concern is that managers value research less than their clinician counterparts. Consequently, research is at times seen as secondary and antagonistic to direct patient care, and hence nurses and midwives are either deterred from undertaking research or undertake research in their own time.

Inclusion of a section within our survey related to translational research added a contemporary dimension to the study not reported in previous studies. These findings indicate an awareness of research as an interdisciplinary team activity strongly associated with practice and policy. Participants were readily able to describe implementation research projects they had participated in or had witnessed leading to significant impact.

### Implications for practice

The successful conduct of translational research requires preparedness at an individual, unit and organizational level, built on a research conducive culture and environment with established linkages, networks and partnerships [16]. With this in mind the study findings will be instrumental in informing initiatives designed to build research capacity and embed a practice-based research culture within Nursing and Midwifery services across the LHD.

Based on the study findings priority will be given to: educating and engaging managers and CNCs; the development of partnerships; mentorships and scholarship programmes; review of existing and potential data collection practices and platforms; and the creation of links with research expertise and support.

We now have a baseline on which to monitor trends that indicate a shift in research culture and focus on research applied to practice within the LHD. The findings provide trend data around workforce and the nature of research activities that nurses and midwives are engaged in. Data that has not been previously available in our LHD. Given the varying degrees of preparedness and agency across sites, research capacity building initiatives and programs will be nuanced to meet individual needs, emphasis will be directed toward supporting research projects that address real-world clinical issues and developing research skills in our nursing and midwifery workforce that enables a culture and program of clinical research.

### Implications for research

With increasing emphasis on research conducted in practice with clinicians and stakeholders there is a need for further research that examines more directly the impact on nursing and midwifery research.

Increasing focus and understanding of translational and implementation process requires the development of tools that focus on these aspects. In particular, future research is required to examine research as an embedded practice and the structural mechanisms that enable it. To this end, we will modify our survey tool for rollout again in 2022.

The networked nature of our health service has enabled the development of team-based research across sites creating links and partnerships that provide support and spread workload. The relevance and impact of nursing and midwifery research will be enhanced through interdisciplinary collaboration, access to supports such as statisticians and health economists through established connections with our local research institute. Strengthening links with universities will facilitate conversations about the nature and content of research training programs and their relevance for clinicians and practice change, and the development of learning modules focused on translational research skills and implementation science.

### Study limitations

Although the sample size was large (816) it is only 10% of the total population of LHD nurses and midwives. However, the data are reasonably representative of the whole LHD cohort, apart from the rural/metro representation. A limitation of the focus groups was that more research related positions are located in metropolitan areas than in rural so there is a risk of metropolitan bias.

## Conclusion

To our knowledge this is the first study to include translational research as a construct in a survey of nurses and midwives research capacity. Our findings suggest that translational research offers nurses a means by which to engage in research in a way that is meaningful to them and their aspirations. Despite variable perceptions regarding the value and relevance of research across the LHD, there are significant numbers of nurses and midwives in our District working across a range of clinical contexts who are well positioned and want to become more involved in research.

Embedding research in nursing and midwifery practice requires greater involvement in multidisciplinary research and more focus on pressing nursing and midwifery issues and concerns. Translational research practice and processes that bring about desired and sustainable change need to be embedded and normalized.

The study findings will inform strategies that recognize and build on increasing interest and preparedness for translational research amongst nurses and midwives in our District.

## Data Availability

The datasets used and/or analysed during the conduct of this study are available from the corresponding author upon reasonable request, and with permission of The Hunter New England Local Health District Nursing and Midwifery Services.

## Abbreviations

LHD: Local Health District
NSW: New South Wales
NMRC: Nursing and Midwifery Research Centre
CNC: Clinical Nurse Consultant
CMC: Clinical Midwifery Consultant
RN: Registered Nurse
CNS: Clinical Nurse Specialist
CMS: Clinical Midwifery Specialist
CNE: Clinical Nurse Educator
